# Supplementary material for: Harnessing changes in open chromatin determined by ATAC-seq to generate insulin-responsive reporter constructs
Source: BMC Genomics. 2022 May 25;23:399. doi: 10.1186/s12864-022-08637-y (PMC9134605; doi:10.1186/s12864-022-08637-y)
Supplement: Supplementary file 1 — Additional file 1. ATAC-seq quality control metrics. [file 12864_2022_8637_MOESM1_ESM.pdf]

A

Sample	Total reads	Uniquely mapped (%)	Multiple mapping (%)	Unmapped (%)
FV1	15,950,956	12,386,888 (77.7)	3,182,285 (20.0)	379,659 (2.4)
FV2	15,103,825	11,769,069 (77.9)	2,963,058 (19.6)	369,650 (2.4)
FV3	19,901,587	15,375,952 (77.3)	4,044,883 (20.3)	477,575 (2.4)
FI1	20,459,629	15,893,639 (77.7)	4,089,116 (20.0)	475,836 (2.3)
FI2	18,610,600	14,482,423 (77.8)	3,677,230 (19.8)	450,208 (2.4)
FI3	17,872,697	13,942,526 (78.0)	3,503,986 (19.6)	425,270 (2.4)

Sample	Total mapped	Nonduplicate count	Duplicate count	Duplication rate
FV1	15,569,173	10,935,840	4,633,333	29.76%
FV2	14,732,127	10,298,933	4,433,194	30.09%
FV3	19,420,835	13,089,863	6,330,972	32.60%
FI1	19,982,755	14,322,959	5,659,796	28.32%
FI2	18,159,653	13,535,781	4,623,872	25.46%
FI3	17,446,512	13,111,747	4,334,765	24.85%

B

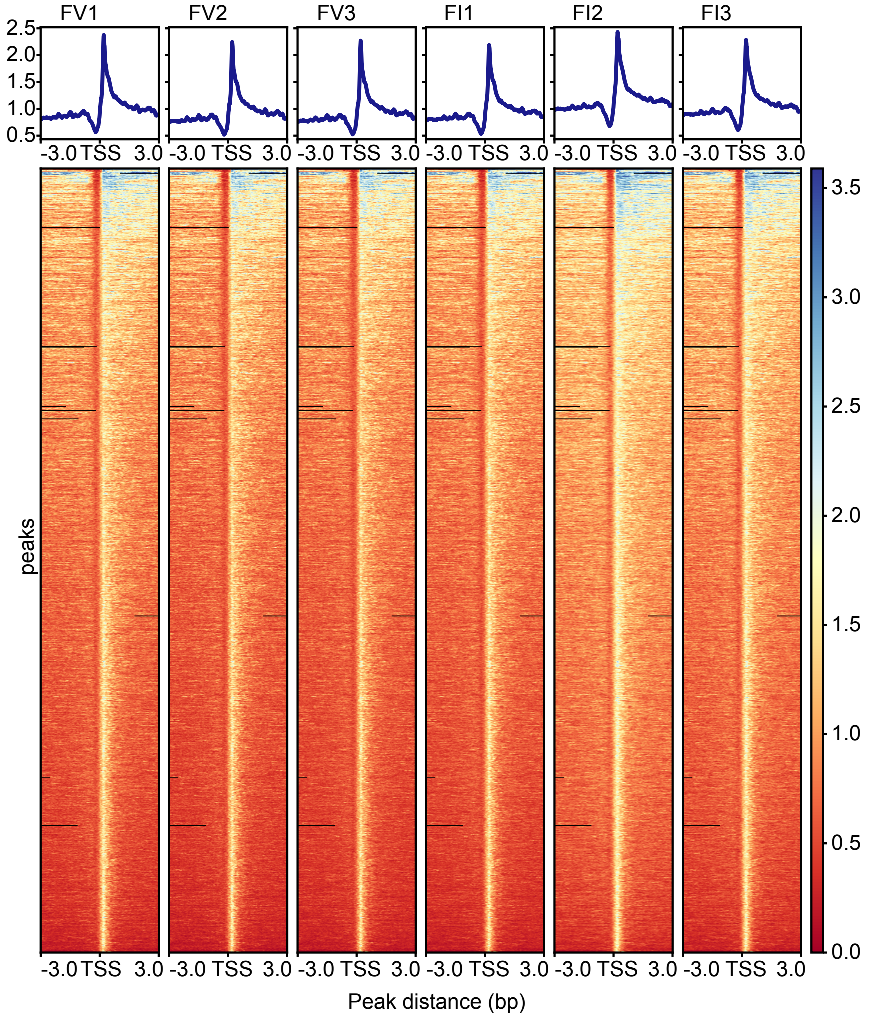

C

Sample	Fraction of reads in peaks (FRiP)
FV1	28.5
FV2	28.5
FV3	27.8
FI1	28.1
FI2	26.4
FI3	27.2

D

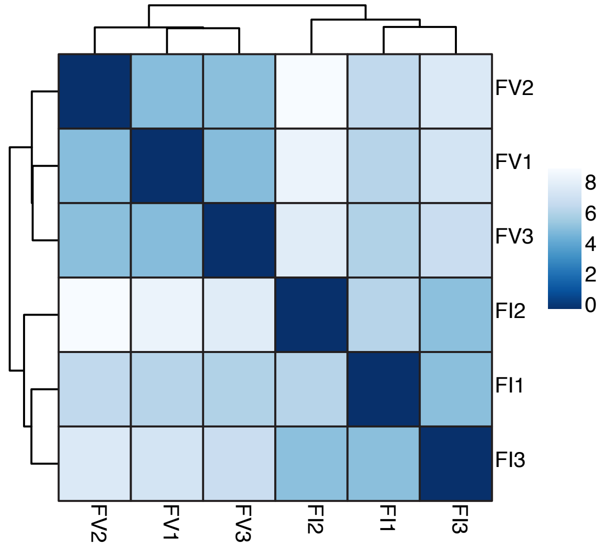

E

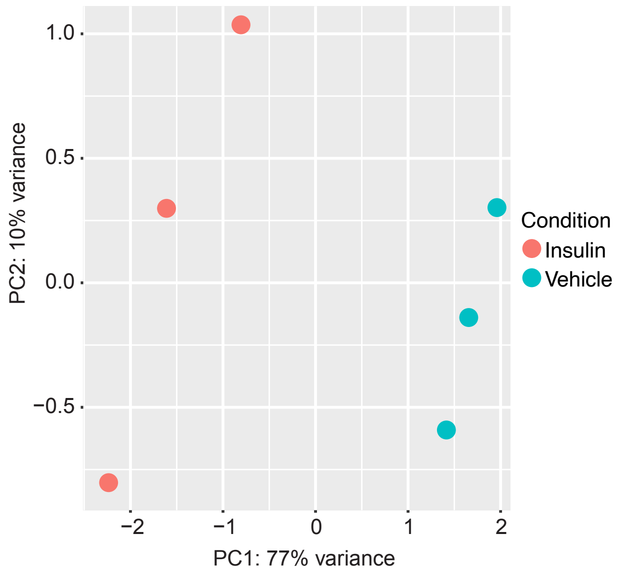

Additional_File_1. Quality metrics of ATAC-seq data. A) Alignment statistics. B) Transcription start site (TSS) enrichment. C) Fraction of reads in peaks. D) Sample similarity calculated by DESeq2. E) Principal component analysis showing sample clustering.
